# Novel multiplex real-time PCR assays reveal a high prevalence of diarrhoeagenic *Escherichia coli* pathotypes in healthy and diarrhoeal children in the south of Vietnam

**DOI:** 10.1186/s12866-020-01878-5

**Published:** 2020-07-03

**Authors:** Vu Thuy Duong, Le Thi Phuong Tu, Ha Thanh Tuyen, Le Thi Quynh Nhi, James I. Campbell, Pham Van Minh, Hoang Le Phuc, Tran Thi Hong Chau, Nguyen Minh Ngoc, Lu Lan Vi, Claire Jenkins, Iruka Okeke, Ellen Higginson, Stephen Baker

**Affiliations:** 1 The Hospital for Tropical Diseases, Wellcome Trust Major Overseas Programme, Oxford University Clinical Research Unit, Ho Chi Minh City, Vietnam; 2 Children’s Hospital 1, Ho Chi Minh City, Vietnam; 3 University of Medicine and Pharmacy at Ho Chi Minh City, Ho Chi Minh City, Vietnam; 4 Children’s Hospital 2, Ho Chi Minh City, Vietnam; 5 The Hospital for Tropical Diseases, Ho Chi Minh City, Vietnam; 6 National Infection Service, Public Health England, England, UK; 7 Department of Pharmaceutical Microbiology, Faculty of Pharmacy, University of Ibadan, Ibadan, Nigeria; 8 Cambridge Institute of Therapeutic Immunology & Infectious Disease (CITIID) Department of Medicine, Cambridge Biomedical Campus, University of Cambridge, Cambridge, CB2 0AW UK

**Keywords:** ETEC, EAEC, EIEC, EPEC, EHEC, Multiplex real-time PCR, Diarrhoea children, Healthy children, Co-infection

## Abstract

**Background:**

Diarrhoeagenic *Escherichia coli* (DEC) infections are common in children in low-middle income countries (LMICs). However, detecting the various DEC pathotypes is complex as they cannot be differentiated by classical microbiology. We developed four multiplex real-time PCR assays were to detect virulence markers of six DEC pathotypes; specificity was tested using DEC controls and other enteric pathogens. PCR amplicons from the six *E. coli* pathotypes were purified and amplified to be used to optimize PCR reactions and to calculate reproducibility. After validation, these assays were applied to clinical samples from healthy and diarrhoeal Vietnamese children and associated with clinical data.

**Results:**

The multiplex real-time PCRs were found to be reproducible, and specific. At least one DEC variant was detected in 34.7% (978/2815) of the faecal samples from diarrhoeal children; EAEC, EIEC and atypical EPEC were most frequent Notably, 41.2% (205/498) of samples from non-diarrhoeal children was positive with a DEC pathotype. In this population, only EIEC, which was detected in 34.3% (99/289) of diarrhoeal samples vs. 0.8% (4/498) non-diarrhoeal samples (*p* < 0.001), was significantly associated with diarrhoea. Multiplex real-time PCR when applied to clinical samples is an efficient and high-throughput approach to DEC pathotypes.

**Conclusions:**

This approach revealed high carriage rates of DEC pathotypes among Vietnamese children. We describe a novel diagnostic approach for DEC, which provides baseline data for future surveillance studies assessing DEC burden in LMICs.

## Background

Diarrhoeal illness remains the second-highest cause of mortality and morbidity worldwide [1–3]; the main burden of this disease occurs in children in South Asia, Southeast Asia, and Africa [3]. Among the bacterial pathogens associated with diarrhoea in children*, Escherichia coli* are repeatedly the most common food borne pathogenic species identified [3–6]. However, identifying diarrhoea-causing *E. coli* can be complex, as pathogenic variants cannot be delineated from commensal *E. coli* solely by microbiological culture.

Diarrhoeagenic *E. coli* (DEC) can generally be divided into six pathotypes (enterotoxigenic *E. coli*, ETEC; enteroaggregative *E. coli*, EAEC; enteropathogenic *E. coli*, EPEC, enteroinvasive *E. coli*, EIEC; enterohemorrhagic *E. coli*, EHEC and shiga-toxin producing *E. coli*, STEC), based on specific virulence markers that are encoded on plasmids and/or chromosomal islands [7]. ETEC, EAEC, and EPEC have all been implicated in causing diarrhoea in young children in low-middle income countries (LMICs) [8–10].

For ETEC, heat-stable toxin producing strains (ST-ETEC) are among the most important pathogens associated with diarrhoea in children [9–11]. Similarly, typical EPEC (possessing both *eae* and *bfp* virulence genes) are more strongly associated with diarrhoea in children in developing regions than atypical EPEC strains which lack *bfp* [6, 12]. EIEC are virtually indistinguishable from *Shigella spp.*, which are essentially an independent genus within the broader *E. coli* population. STEC are commonly associated with food-borne disease outbreaks in developed countries and have higher mortality than other *E. coli* pathotypes due to sequelae of haemolytic uraemic syndrome (HUS) [13–15]. The epidemiology of STEC in LMICs, particularly in children in Southeast Asia, are not well described.

The proportion of diarrhoeal disease associated with DEC in Vietnam is not well investigated as measuring the prevalence of these pathogens in diarrhoeal cases and non-diarrhoeal controls is laborious and not routinely performed. Of the limited DEC studies conducted in Vietnam an investigation originating in Hanoi detected DEC in 22% of stool sample from diarrhoeal cases and 12% of controls using conventional multiplex PCR [16]. Here, we aimed to develop a set of standardized multiplex real-time PCR assays to identify the various DEC in complex samples in a comparatively short turnaround time. To establish the multiplex real-time PCR assays to identify the six DEC pathotypes we designed new or adapted existing specific primers and probes for nine DEC associated genes. The real-time PCR assays were optimized and then used to determine the prevalence of DEC in children with and without diarrhoea disease in Ho Chi Minh City (HCMC), Vietnam. Lastly, we combined these PCR data with available clinical data to identify clinical features in children infected with differing DEC pathotypes and to determine the potential effect of DEC in the stools of diseased and non-diseased children.

## Results

### Multiplex real-time PCR assay for detecting diarrhoeagenic *Escherichia coli*

We firstly validated PCR amplification for ETEC, EAEC, EIEC*/Shigella*, EPEC, and STEC in monoplex using cloned target sequences and then with genomic DNA extracted from the various *E. coli* pathovars. The sensitivity of the primer and probe sets was determined by generating a series of standard curves using 10-fold dilutions of control plasmid DNA. The limit of detection for all targets, including the *uidA* control, was five copies per reaction, with the exception of *aggR* which could be detected down to 50 copies per reaction. Each primer and probe set were tested against a panel of commonly isolated pathogens found in stool samples, which included *Staphylococcus aureus*, *Klebsiella pneumoniae*, *Salmonella spp*., *Campylobacter coli*, *Campylobacter jejuni*, *Shigella sonnei*, *Shigella flexneri*, *Enterobacter*, *Proteus*, norovirus, and rotavirus (these viruses were selected as they are most commonly found viruses in the stools of children with diarrhoea). No amplification was observed in any sample other than those containing *E. coli*.

Ultimately, the PCR assays were multiplexed into four reactions, and the sensitivity, intra-assay and inter-assay CVs across the nine target sequences were calculated for each multiplexed PCR reaction. The Ct values for each target were equivalent between the monoplex and multiplex reactions, confirming that multiplexing did not impact sensitivity. The intra-assay and inter-assay CVs ranged from 0.01 to 1.54% and from 0.01 to 2.12%, respectively (Table 1). The linear regressions of the standard curves were between 0.992–0.999 for all targets tested. The resulting efficiency of the amplification ranged from 90.9 to 105.7%, demonstrating the multiplex real-time PCR assays were well optimized, reproducible, and specific.

**Table 1:** Reproducibility of the assays on diluted plasmid DNA containing cloned target sequences

Target sequence	Co-efficient of variance (%)	Target concentration							
		5 × 10^7^	5 × 10^6^	5 × 10^5^	5 × 10^4^	5 × 10^3^	5 × 10^2^	5 × 10^1^	5 × 10^0^
*uidA*	Intra-assay variation ^a^	0.13	0.09	0.14	0.26	0.34	0.12	0.82	0.52
	Inter-assay variation ^b^	0.18	0.18	0.21	0.73	0.66	0.74	0.66	0.93
*eltB*	Intra-assay variation	0.03	0.04	0.06	0.10	0.35	0.16	0.27	0.35
	Inter-assay variation	0.07	0.11	0.05	0.23	0.41	0.50	0.38	0.67
*estA*	Intra-assay variation	0.01	0.05	0.03	0.25	0.19	0.16	0.15	0.06
	Inter-assay variation	0.15	0.03	0.04	0.05	0.52	0.36	0.12	0.76
*aggR*	Intra-assay variation	0.11	0.08	0.08	0.15	0.20	0.37	0.29	–
	Inter-assay variation	0.06	0.11	0.33	0.03	0.29	0.23	1.05	–
*ipaH*	Intra-assay variation	0.19	0.17	0.20	0.12	0.12	0.60	0.15	0.11
	Inter-assay variation	0.23	0.28	0.20	0.07	0.42	0.20	0.52	1.42
*eae*	Intra-assay variation	0.07	0.24	0.29	0.06	0.26	0.76	0.20	0.48
	Inter-assay variation	0.38	0.16	0.06	0.46	0.82	0.64	0.63	0.88
*bfpA*	Intra-assay variation	0.03	0.09	0.11	0.17	0.50	0.90	0.63	0.90
	Inter-assay variation	0.01	0.11	0.05	0.06	0.18	0.38	0.75	1.23
*rfbE*	Intra-assay variation	0.03	0.11	0.09	0.42	0.20	0.70	0.98	0.30
	Inter-assay variation	0.02	0.04	0.10	0.23	0.60	1.49	1.29	1.08
*stx1*	Intra-assay variation	0.13	0.13	0.17	0.21	0.50	0.74	0.97	0.60
	Inter-assay variation	0.22	0.50	0.71	0.98	1.19	1.57	1.95	0.55
*stx2*	Intra-assay variation	0.11	0.14	0.18	0.17	0.25	1.54	0.81	0.24
	Inter-assay variation	0.12	0.22	0.43	0.47	0.87	1.54	2.12	1.97

^a^ Intra-assay variation was calculated by measuring the co-efficient of variance of the Ct value on three concurrently run assays

^b^ Inter-assay variation was calculated by comparing variation in Ct value on three independently run assays

### The prevalence of diarrhoeagenic *Escherichia coli* from faecal specimens of children hospitalized with diarrhoea

Between May 2014 and April 2016, we amassed 2815 MC sweeps (i.e. faecal samples plated on MC media) from 3166 children hospitalized with bloody and/or mucoid diarrhoea at three tertiary hospitals in HCMC. A single faecal sample was collected from each child within their first 2 days of hospital admission for diarrhoea. The majority of patients were male (1731/2815; 61.5%), with ages ranging from one month to 15 years (median age 10 months, IQR 6.6–17.1 months).

We employed the four multiplex real-time DEC PCRs on all 2815 MC sweeps to identify DEC targets potentially associated with clinical infection. At least one PCR amplification associated with a DEC variant was positive in 34.7% (978/2815) of the MC sweeps from paediatric patients hospitalized with diarrhoea. Among the DEC amplification positive samples, EAEC was the most common variant detected, with *aggR* amplified in 15.7% (443/2815) of samples (Table 2). Other commonly amplified DEC targets included EIEC/*Shigella* and EPEC, which were identified in 12.4% (349/2815) and 12.2% (343/2815) of the MC sweep samples, respectively.

**Table 2:** DEC detected in children hospitalized with diarrhoea (*N* = 2815)

Pathotypes	Target gene	N	%
ETEC		182	6.5
LT-ETEC	*elt*	167	5.9
ST-ETEC	*est*	7	0.2
LT-ST-ETEC	*elt* & *est*	8	0.3
EAEC	*aggR*	443	15.7
EIEC/*Shigella*	*ipa*H	349	12.4
EPEC		343	12.2
Atypical EPEC	*eae*	322	11.4
Typical EPEC	*eae* & *bfpA*	21	0.7
EHEC/STEC		46	1.6
O157	*eae* & *rfbE_O157*	20	0.7
non-eae O157	*rfbE_O157*	26	0.9
	*stx1*/s*tx2*	4	0.1
Negative		1837	65.3

Within the EPEC pathotype, atypical EPEC positive samples (*eae* positive, *bfpA* negative) were more prevalent than typical EPEC positive samples (*eae* positive, *bfpA* positive); 93.9% (322/343) vs. 6.1% (21/343), respectively. ETEC was detected in 6% (182/2815) of samples, with only a limited number of these samples (8.2%; 15/182) producing an amplicon for heat stable toxin (*estA).* Four diarrhoeal patients harboured samples containing the Shiga toxin-producing genes (*stx1*/*stx2*). Among the four cases associated with an STEC positive sample, one was positive for *eae* and one was positive for both *eae* and *rfbE_O157*. Of the two STEC cases that were amplification positive for *eae* and *rfbE_O157*, one was additionally positive for *eltB* (ETEC), the other was positive for *aggR* (EAEC).

### Clinical manifestations of diarrhoeagenic *Escherichia coli* mono-infection

To investigate clinical syndromes associated with the various DEC in Vietnam, clinical data associated with the patients were accessed and compared between pathotype groups (Table S2). Patient samples from which multiple DEC pathogens were amplified were excluded. Notably, ~ 70% of those with an ETEC, EAEC, EPEC, or STEC O157 positive sample were associated with mucoid, non-bloody diarrhoea, whereas EIEC/*Shigella* was significantly associated with visible bloody diarrhoea (39.7%, 46/116, *p* < 0.001, χ^2^ test). EAEC was the most commonly identified DEC in mono-infection. This pathotype was more commonly associated with children that had wasting or severe wasting (13.5%, 23/170; *p* = 0.013, Fisher’s exact test) than the other DEC variants. Whilst EHEC_O157 was identified less frequently than other pathotypes, it was significantly associated with moderate and severe dehydration (40%, 8/20; *p* = 0.010, Fisher’s exact test), which commonly required intravenous rehydration therapy.

Generally, we found that infections associated with DEC positive samples were uncomplicated; > 90% of patients had improved or recovered after 3 days and their median hospital stay was 5 days [IQR 3–7 days]. The use of antimicrobials within this study population was high, with 81.3% (1513/1861) of patients receiving empirical antimicrobial treatment prior to any diagnostic testing, which may impact on the detection of various DEC, depending on their susceptibility profile. Fluoroquinolones, specifically ciprofloxacin, were the most commonly used class of antimicrobials in those with a DEC in their stool (957/1512, 63.3%).

### Diarrhoeagenic *Escherichia coli* from faecal specimens of diarrhoeal hospitalized children vs. healthy non-diarrhoeal children

Between March 2016 and August 2016, 498 MC sweeps were additionally collected from faecal samples taken from healthy children residing in HCMC and participating in a cohort study [17]. The majority of healthy children were male (269/498; 54.0%), with their age when sampled ranging from 24 months to 5 years (median age 46.4 months, IQR 35.6–52.5 months). In a comparable manner to the diarrhoeal samples, we screened the MC extractions from these healthy children with the multiplex real-time PCRs to detect DEC. At least one pathotype of DEC was detected in 41.2% (205/498) of samples associated with non-diarrhoeal children (Table 3).

**Table 3:** Direct comparison of DEC detected in samples from children hospitalized with diarrhoea and healthy children

Diarrhoeagenic ***E. coli***	Target gene	Diarrhoea N (%)		Non-diarrhoea N (%)		***p*** value*
Number		319		498		
ETEC		29	(9.1)	42	(8.4)	0.745
LT-ETEC	*elt*	25	(7.8)	39	(7.8)	0.998
ST-ETEC	*est*	1	(0.3)	3	(0.6)	1.000
LT-ST-ETEC	*elt* & *est*	3	(0.9)	0	(0.0)	0.059
EAEC	*aggR*	50	(15.7)	89	(17.9)	0.415
EIEC/*Shigella*	*ipa*H	93	(29.2)	4	(0.8)	**< 0.001**
EPEC		39	(12.2)	93	(18.7)	**0.015**
Atypical EPEC	*eae*	38	(11.9)	90	(18.1)	**0.018**
Typical EPEC	*eae* & *bfpA*	1	(0.3)	3	(0.6)	1.000
EHEC/STEC		13	(4.1)	20	(4.0)	0.712
O157	*eae* & *rfbE_O157*	5	(1.6)	5	(0.6)	0.524
non-eae O157	*rfbE_O157*	8	(2.5)	18	(3.4)	0.379
	*stx1*/*stx2*	0	–	7	(1.4)	**0.033**
Negative		163	(51.1)	293	(58.8)	**0.030**

*p value from χ^2^ test or *Fisher’s exact* test

To determine the prevalence and distribution of the various DEC in healthy and diarrhoeal children, we compared the data from the healthy children with a subset of the data from matched children in the diarrhoeal study which were between the ages of 2 and 5 years old (319 children; median age 31.5 months, IQR 26.7–38.9 months). The prevalence of ETEC, EAEC, and EHEC_O157 in faecal samples was not significantly different between children with or without diarrhoea (Table 3, Fig. 2). Furthermore, EPEC was detected significantly more frequently in the non-diarrhoeal samples (18.7%, 93/498) than the diarrhoeal samples (11.4%, 33/289) (*p* = 0.019, χ^2^ test) (Table 3). The only DEC that was significantly associated with the diarrhoeal samples was EIEC/*Shigella*, which was detected in 34.3% (99/289) of diarrhoeal samples vs. 0.8% (4/498) non-diarrhoeal samples (*p* < 0.001, Fisher’s exact test).

The distribution of DEC co-infection among the cases and the controls was complex and highly variable (Fig. 2). The most common co-infections in the diarrhoeal group were EAEC + EIEC/*Shigella* (3.8%, 12/319) and EAEC + EIEC/*Shigella* + ETEC (2.2%, 7/319); whereas EPEC + EAEC (3.4%, 17/498) was more common in the healthy control group. Co-infection with more than one DEC was more likely to be associated with diarrhoeal disease than with healthy controls (16.3%, 52/319 vs. 9.6%, 48/498, *p* = 0.005, χ^2^ test). However, due to the predominant presence of EIEC/*Shigella* in the diarrhoeal group, EIEC/*Shigella* infection was a potential confounder.

To disaggregate the potential confounding effect of EIEC/*Shigella*, we performed binary univariate and multivariate logistic regression to identify variables and DEC that were associated with diarrhoeal disease in children aged 24–60 months (Table 4). In the univariate model, co-infection with ETEC, mono-infection with EIEC/*Shigella*, co-infection with EIEC/*Shigella*, and co-infection without EPEC, EHEC_O157, and STEC were significantly associated with diarrhoea. However, after controlling for confounders, only mono or co-infection with EIEC/*Shigella* and wasting were determined to be significantly associated with diarrhoea. Conversely, mono-infection with ETEC, EAEC, and obesity were significantly more common in the non-diarrhoeal children.

**Table 4:** Univariate and multivariate analysis of DEC mono-infection and co-infection associated with diarrhoeal disease among children from 24 to 60 months of age using binary logistic regression model

Variable	Univariate Model			Multivariate Model		
	Odds Ratio	95% Confidence Interval	***P***-value^c^	Odds Ratio	95% Confidence Interval	***P***-value^d^
Types of infection ^a^						
Mono-infection with ETEC	0.39	0.15–1.05	0.062	0.32	0.11–0.94	**0.037**
Co-infection with ETEC	2.27	1.21–4.27	**0.011**			
Co-infection without ETEC	1.74	1.00–3.02	0.051			
Mono-infection with EAEC	0.51	0.28–0.93	0.028	0.45	0.24–0.86	**0.015**
Co-infection with EAEC	1.75	1.06–2.89	0.030			
Co-infection without EAEC	2.55	1.19–5.46	0.016			
Mono-infection with EIEC	52.13	12.57–216.21	**< 0.001**	49.66	11.90–207.24	**< 0.001**
Co-infection with EIEC	31.46	7.47–132.47	**< 0.001**	35.60	8.28–153.12	**< 0.001**
Co-infection without EIEC	0.66	0.37–1.20	0.173	0.63	0.34–1.18	0.149
Mono-infection with EPEC	0.55	0.32–0.95	0.031	0.59	0.34–1.02	0.057
Co-infection with EPEC	1.22	0.66–2.25	0.525			
Co-infection without EPEC	2.97	1.65–5.34	**< 0.001**			
Mono-infection with EHEC	0.90	0.33–2.44	0.834	0.96	0.35–2.64	0.937
Co-infection with EHEC	1.14	0.44–3.01	0.785			
Co-infection without EHEC	2.19	1.36–3.52	**0.001**			
Mono-infection with STEC	–					
Co-infection with STEC	–					
Co-infection without STEC	2.17	1.39–3.40	**0.001**			
Gender						
Female	0.81	0.61–1.07	0.142	0.65	0.47–0.91	**0.013**
Growth ^b^						
Obese	0.57	0.35–0.92	0.022	0.51	0.29–0.89	**0.019**
Overweight	0.88	0.57–1.36	0.560	1.00	0.62–1.62	0.998
Risk of overweight	0.68	0.46–1.01	0.053	0.83	0.54–1.27	0.390
Wasted	19.13	2.48–147.54	**0.005**	19.58	2.43–157.92	**0.005**

^a^ Odds Ratio with the reference was non-infection status

^b^ Odds Ratio with the reference was normal growth status

^c^*P*-value considered significant when p < 0.01

^d^*P*-value considered significant when *p* < 0.05

## Discussion

Here, we developed and applied an efficient and robust collection of real-time PCR assays for identifying DEC in MC sweeps isolated from stool samples from a collection of healthy and diarrhoeal children. This approach, in comparison to the traditional method, is straightforward, cost-effective and has a comparatively short turn-around time [18]. Ultimately, the four multiplex real-time PCR assays could detect ten target sequences corresponding with six pathotypes of DEC, which permitted detection of these pathogens with a high degree of accuracy and utility. However, there are some limitations with our approach. Due to their high genetic similarity, we are unable to differentiate between EIEC and *Shigella spp.* by using real-time PCR, as the invasion plasmid antigen H (*ipaH*) and the *uidA* (the internal control gene for *E. coli*) are present in both [19]. Further limitations of this approach are associated with issues of how pathotypes such as EPEC, EHEC, and STEC are defined. Through bacterial genomics, we know that organisms lacking either *eae* or *stx* or both may still belong to the EHEC group [20, 21]. In addition, the *stx* genes have been found in other pathotypes of *E. coli* [22, 23]. Therefore, it is impossible to definitively assign an *E. coli* to a DEC pathotype without genome sequencing. However, pathotyping DEC through detecting virulence genes remains useful for assessing the potential prevalence of the various pathogenic forms of *E. coli* in any given population. In addition to the methodological constraints of the study, as our control samples came from healthy children over 12 months of age, we could not evaluate associations with diarrhoea in children under 1 year of age and we recognise that co-infection with organisms that were not detected may impact on disease presentation.

While ETEC is the most common DEC internationally, the prevalence of ETEC in this setting was found to be considerably lower than other regions [9, 18]. This result is probably due to the study inclusion criteria, as only children presenting with bloody and/or mucoid diarrhoeal illness were enrolled, whereas ETEC is most commonly associated with watery diarrhoea [7]. Here, LT-ETEC were more prevalent than ST-ETEC, which is consistent with earlier studies on ETEC infections in children. However, in these previous studies the association between LT-ETEC infection and diarrhoea was weak [6, 9, 18]. In contrast, in the Global Enteric Multicentre Study (GEMS), ST-ETEC but not LT-ETEC was attributed as a major cause of diarrhoea in all age groups [24]. To determine whether ST-ETEC is an important pathogen in Vietnam, it will be necessary to carry out additional studies focusing on children presenting with watery diarrhoea.

EAEC was the most commonly detected pathotype in children with diarrhoea in this study, which is again consistent with earlier studies that reported high detection rates of EAEC compared to other DEC in Vietnam [16, 25]. Several articles have raised the possibility that not all EAEC are pathogenic, and that variants within this group may have different propensities to cause disease [26–31]. However, several outbreaks and human volunteer studies have unequivocally shown that some EAEC can cause disease [26–31]. Here, one third of EAEC mono-infections required antimicrobial IV treatment (i.e. the third generation cephalosporins or imipenem; data not shown) associated with a more severe disease presentation. Notably, samples from three children in this study generated positive PCR amplicons for both EAEC and *stx*. These cases may represent mixed infections of EAEC and STEC, or potentially hybrid organisms, such as those associated with an extensive outbreak in Europe in 2011 [23]. Although EAEC was not associated with diarrhoea in children within the 24–60-month age group in this study, it was the most commonly detected pathotype from children with wasting. This observation is consistent with the findings of the recent the MAL-ED study, which reported that EAEC infection is associated with growth shortfall, irrespective of disease [32].

EPEC was the most common DEC gene target amplified from faecal samples of diarrhoea and non-diarrhoea children. The overwhelming majority of the amplicons generated from both healthy and diseased cohorts were associated with aEPEC. These data are again consistent with EPEC literature, which suggests that typical EPEC is commonly identified in the African continent [18, 33], while atypical EPEC tends to predominate in other regions [34]. A case-control study conducted in seven LMICs found that typical EPEC infections were significantly associated with mortality in children under 5 years [6]. The high prevalence of atypical EPEC positive samples in our study group (24–60 months of age) may be partially associated with colonization in the first year of life, as asymptomatic infection with ETEC, EAEC, and EPEC have previously been associated with weaning and the termination of breastfeeding [35].

STEC O157 cause severe diarrhoea and are associated with a high mortality rate in food-borne outbreaks in western countries [36]. EHEC_O157 in this setting had a low prevalence and more than half the positive samples were positive for the *rfbE_O157* gene alone, which suggests these are likely to be of lower pathogenicity. Only two samples that tested positive for EHEC_O157 also produced amplicons for the Shiga-toxin gene (*stx2*), suggesting that O157-STEC is not a significant cause of gastrointestinal symptoms in this location. In the age matched comparison, STEC were isolated from children in the healthy group only. This observation is consistent with data originating in Indonesia, where STEC was detected significantly more frequently in non-diarrhoeal children [37].

In previous studies, co-infection with more than one DEC (or with other enteric pathogens) was found to be significantly associated with diarrhoea [26, 38–41]. In this study, we found that co-infection with DEC was not associated with diarrhoea and was also common in healthy children. Notably, only co-infection with EIEC/*Shigella* was significantly associated with diarrhoeal disease. However, as EIEC/*Shigella* infection alone was highly significantly associated with diarrhoeal illness, the contribution of other DEC to disease in EIEC/*Shigella* infection is unclear. In a multivariate logistic regression model, DEC co-infection in the absence of EIEC/*Shigella* was not associated with diarrhoea. This suggests that EIEC/*Shigella* is the most important cause of DEC mediated moderate-to-severe diarrhoea in this setting.

## Conclusions

Multiplex real-time PCR is an efficient method for detecting the six major pathotypes of DEC in a collection of clinical samples. This new methodology provides a useful alternative to classical microbiology for large-scale microbiological and epidemiological studies. Using this approach, we found a high prevalence of DEC in the stools of both healthy and diarrhoeal children in Vietnam. EAEC and atypical EPEC were the most commonly detected DEC in both groups; whereas, EIEC/*Shigella* was the only DEC significantly associated with diarrhoeal disease. This study provides new methodology and baseline data for further clinical, epidemiological, and genomic studies in Vietnam and across Southeast Asia and shows that DEC are highly prevalent but not generally associated with diarrhoeal disease in Vietnam.

## Methods

### Study design

Children aged ≤15 years with diarrhoeal illness admitted to one of the three collaborating tertiary hospitals in HCMC, Vietnam from May 2014 to April 2016 were eligible for enrolment. Those with diarrhoeal illness (cases) were defined as ≥3 passages of loose stools within 24-h period along with at least one loose stool containing blood and/or mucus [42]. We excluded children if they had suspected or confirmed intussusception at the time of enrolment [43]. Controls were healthy children between the age of 12–60 months enrolled in diarrhoeal disease cohort in District 8 in HCMC from 2014 to 2016 [17]. The enrolled children attended HVH for routine health check every six months. An anal swab of healthy child was collected by study nurses at these routine visits.

### Primer and probe design

**Fig. 1:**
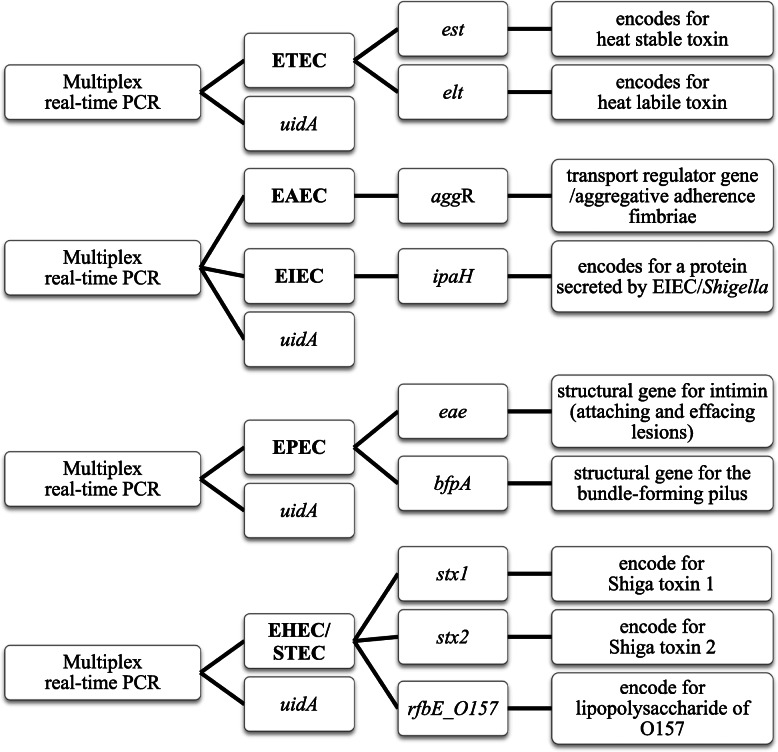
Multiplex PCR strategy. Flowchart showing the four multiplex real-time PCR assays for detecting target sequences of DEC; *uidA* (gene encoded for β-glucuronidase and presented in all *E. coli*) was selected as an internal control

The selected target genes for each pathotype were: ETEC, *eltB* (heat-labile toxin) and/or *estA* (heat-stable toxin); EAEC, *aggR* (transport regulator gene [44]); EIEC, *ipaH* (secreted protein encoded on pINV [19]); EPEC, *eae* (encoding the intimin adherence gene [7]) and *bfpA* (encoding a structural component of the bundle forming pilus [45]); STEC, *stx1* and/or *stx2* (Shiga toxins [7]); and *rfbE_O157* (encoding the lipopolysaccharide O157 antigen, the most common STEC serogroup in regions where surveillance data is available). The *uidA* gene, which encodes beta-glucuronidase and is present in all *E. coli*, was used as an internal control to monitor both DNA extraction and PCR amplification. A flowchart of the combined assay strategy is shown in Fig. 1.

We classified the DEC amplification results using the following approach; ETEC positive samples were divided into LT-ETEC (*eltB* positive only); ST-ETEC (*estA* positive only); and LT-ST-ETEC (*eltB* and *estA* positive). Amplification of *aggR* was sufficient for classification as EAEC and a positive amplification for *ipaH* identified EIEC/*Shigella*. EPEC positive samples were divided into typical EPEC (carrying both *eae* and *bfpA)* and atypical EPEC (the presence of *eae* only). The STEC pathotype was identified by the presence of stx1 and/or stx2, and the presence or absence of *rfb_O157* was used to differentiate between STEC O157 and the non-O157 STEC serogroups. STEC that have the potential to cause HUS carry additional virulence genes, specifically *eae* and *aggR*.

The primer and probe sequences for *aggR*, *ipaH*, *eae*, and *uidA*) were adapted from previous studies [46–49]. Reference sequences were downloaded from GenBank *eltB*, *estA (STh)*, *bfpA*, *rfbE_O157*, *stx1* and *stx2* (Table S1) and aligned using AlignX (Vector NTI, Invitrogen) to identify conserved regions within the gene sequence. Primer Quest (IDT, USA) was employed to generate primers and probes of amplicon size 100 to 150 bp. To find optimal pairs, candidate primers and probes were analysed for Tm, %GC, hairpin, self-dimer and hetero-dimer using Oligo Analyzer 3.1 (http://sg.idtdna.com/calc/analyzer). Final primer/probe candidates were blasted against PrimerBLAST (https://www.ncbi.nlm.nih.gov/tools/primer-blast/) to confirm the *in-silico* specificity of the selected sequences. The selected primer and probe sequences are shown in Table 5.

**Table 5:** Sequences of primers and probes to detect DEC in this study

Diarrhoeagenic ***E. coli*** target	Primer/probe name	Sequence * (5′-3′)	Amplicon size (bp)
ETEC	*elt*_ETEC_F	CTCGGTCAGATATGYGATTCTT	100
	*elt*_ETEC_R	AACATTTCAGGTCGAAGTCC	
	*elt*_ETEC_ probe	**FAM**-TGTGTCCTTCATCCTTTC AATGGCTT-**BHQ1**	
	*est*_ETEC_F	GCTAAACCAGYAGRGTCTTCAA	137
	*est*_ETEC_R	GCAGGATTACAACACAATTCAC	
	*est*_ETEC_ probe	**LCCyan500**-AGTRGTCCTGAAA GCATGAATAGTAGCA-**BHQ1**	
EAEC	*aggR*_F	CCATTTATCGCAATCAGATTAA	92 [46]
	*aggR*_R	CAAGCATCTACTTTTGATATTCC	
	*aggR*_probe	**FAM**-CAGCGATACATTAAGA CGCCTAAAGGA-**BHQ1**	
EIEC/*Shigella*	*ipaH*_F	AGGTCGCTGCATGGCTGGAA	99 [47]
	*ipaH*_R	CACGGTCCTCACAGCTCTCA	
	*ipaH*_probe	**LCCyan500**-AACTCAGTGCCTCT GCGGAGCTTCGACA-**BHQ1**	
EPEC/EHEC	*eae*_F	CATTGATCAGGATTTTTCTGGTGA TA	102 [48]
	*eae*_R	CTCATGCGGAAATAGCCGTTA	
	*eae*_probe	**FAM**-ATAGTCTCGCCAGTA TTCGCCACCAATACC-**BHQ1**	
EPEC	*bfpA*_EPEC_	GTCTRTCTTTGATTGAATCKGC	108
	*bfpA*_EPEC_R	CATTCTGYGMCTTATTGGAATC	
	*bfpA*_EPEC_probe	**LCCyan500**-ACCGTTACYGCM GGTGTGATGTTT-**BHQ1**	
STEC	*stx1*_EHEC_F	GCATCTGATGAGTTTCCTTCTA	113
	*stx1*_EHEC_R	GTTCTGCGCATCAGAATTG	
	*stx1*_EHEC_probe	**FAM**-AAGAGKCCGTGGGA TTACGCACAAT-**BHQ1**	
	*stx2*_EHEC_F	ACRACGGACAGCAGYTATWC	111
	*stx2*_EHEC_R	GAACTCCATTAAMKCCAGATA	
	*stx2*_EHEC_probe	**LC Red 610**-ATGCAAATCAGTCGTCA CTCACTGGT-**BHQ1**	
EHE CO157	*rfbE*_O157_F	CAAGTCCACAAGGAAAGTAAAG	111
	*rfbE*_O157_R	GAGTTTATCTGCAAGGTGATTC	
	*rfbE*_O157_probe	**LCCyan500**-AACTCAGTGCCTCT GCGGAGCTTCGACA-**BHQ1**	
Internal control	*uidA*_F	GTGTGATATCTACCCGCTTCGC	82 [49]
	*uidA*_R	AGAACGGTTTGTGGTTAATCAGGA	
	*uidA*_probe	**CY5**-TCGGCATCCGGTCAGTGGCAGT-**BHQ2**	

*R (A/G), Y(C/T), S (G/C), W (A/T), M (A/C), K (G/T) according to International Union of Pure and Applied Chemistry (IUPAC)

Probe detection format (Roche Light Cycler II 480) as followed **FAM**: 498–580; LC**Cyan500**: 440–488; **CY5**: 618–660; **LC Red 610**: 533–610; BlackBerry® Quencher: **BHQ1**, **BHQ2**

### Isolation of nucleic acids and construction of control plasmids

Nucleic acids were purified from prototypic *E. coli* strains and a variety of other gastrointestinal pathogens using Wizard Genomic DNA Purification Kit (Promega). PCR amplicons were generated for each of the 11 target genes and ligated into pCR™ 2.1-TOPO® (Invitrogen, Applied Biosystem, UK). Purified plasmids were used as template to optimize PCR reactions and measure assay reproducibility. Plasmid concentrations (ng/μl) were quantified using a Nanodrop spectrophotometer (Thermo-Scientific, UK), and converted to copy number using the URI Genomics and Sequencing Center online tool (http://cels.uri.edu/gsc/cndna.html).

### Real-time PCR

Multiplex real-time PCR reactions were performed in a 25 μl reaction mixture containing a final concentration of 1X buffer, 0.2 mM deoxynucleoside triphosphates (dNTPs), 3.5 mM of MgCl_2_, 0.2 μM of each forward and reverse primers, 0.08 μM of each probe and 1 U of Hotstart Taq polymerase (QIAGEN, Germany). Five μl of DNA template was used for each PCR reaction. The real-time PCR cycling conditions were as follows: 95 °C for 15 min, followed by 45 cycles of 95 °C for 15 s, then 60 °C for 60s, using the Light Cycler 480 II system (Roche, Germany). The threshold cycle (Ct) value for a positive result was considered to be 38 or less.

### Reproducibility and linearity analysis

The precision and reproducibility of the real-time PCR assays were assessed using the co-efficient of variance (CV%), measured by dividing the standard deviations of the Ct values by the mean Ct values for each selected concentration. The Ct values of three replicates assayed simultaneously were compared to measure intra-assay reproducibility. The inter-assay reproducibility was calculated from data generated on three separate days. Linearity was determined by linear regression, using Ct values produced from 10-fold dilutions of control plasmid DNA.

### Specimen culture and storage

Diarrhoeal faecal specimens were collected in sterile containers and transported to the laboratory within 24 h [43]. Anal swabs from non-diarrhoeal children were also transported to the laboratory within 24 h for processing. Specimens were inoculated onto MacConkey agar (MC, Oxoid), and incubated at 37 °C for 18–24 h [43]. Following incubation, a sweep of colonies was taken from the entire MC agar plate and suspended in 20% glycerol in Brain Heart Infusion (BHI) broth, before being stored at − 80 °C.

### Crude DNA extraction

Eighty μl of the stored colony sweep suspension was centrifuged at 4000 rpm for 10 min, and the pellet was resuspended in 80 μl of molecular grade water (Sigma). The resulting suspension was mixed by gently pipetting up and down, before being boiled at 96 °C for 10 min and cooled to room temperature. The lysate was centrifuged at 4000 rpm for 10 min to remove cellular debris, and 5 μl of supernatant was subjected to the real-time PCR assays.

### Data collection and statistical analysis

**Fig. 2:**
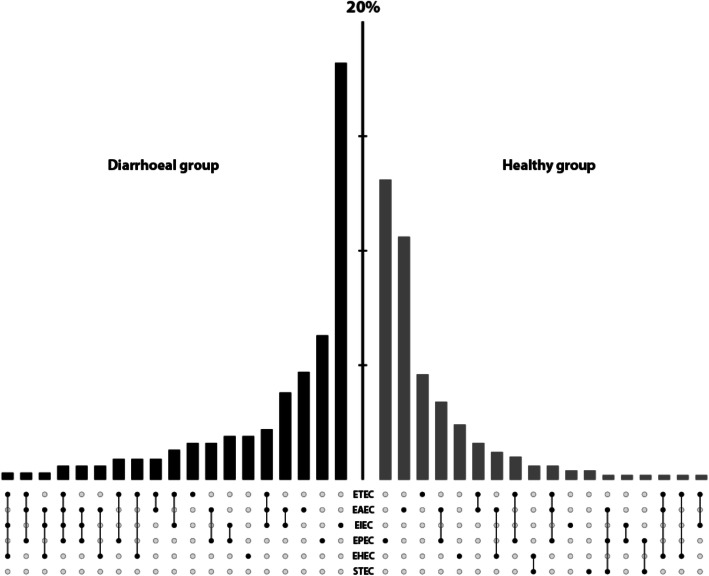
Mono- and co-infection with DEC in the 24–60-month-old healthy and diarrhoeal and healthy children. Bar chart demonstrating the proportion of cases and controls with each combination of DEC pathotypes, with the most frequently isolated DEC pairings located near the centre, the scale (y axis) in 5% increments. The dots and lines between dots at the base of the chart show the co-infection status for six pathotypes of DEC. DEC co-infection patterns among diarrhoea group (*N* = 319; black) and healthy group (*N* = 498; grey)

Data were exported into Microsoft Excel (Microsoft, USA), and analysed using Stata v11 (StataCorp, College Station TX, USA). Descriptive comparisons between groups were conducted using non-parametric tests including χ^2^ test or Fisher’s exact test for categorical variables and the Kruskal-Wallis test for continuous data. Growth status of participating patients were assessed using the WHO global database on growth and nutrition and Prevention and Management of Obesity for Children and Adolescents-Healthcare guidelines using the macro package of Stata v11 developed by WHO [50, 51]. Due to the age difference between diarrhoeal and non-diarrhoeal groups, the comparative analyses were performed between all the children in healthy group and the subset children in the diarrhoea group that were aged 24–60 months. Logistic regression was performed to determine the associations with diarrhoea using each type of infection considered as an independent variable. Infections were classified as mono-infection of each pathotype of DEC or co-infections of each specific pathotype and other pathotypes. The types of co-infection were repeated due to multi pathotype co-infection; hence the *p*-value for univariate model was considered significant when *p* < 0.01. Multivariable logistic regression models were performed and incorporated mono-infections, each specific type of co-infection, gender and growth status with diarrhoea and non-diarrhoea as binary outcomes (performed on Stata v11, StataCorp, College Station TX, USA). For the latter, a *p*-value of < 0.05 was considered significant. The figure for mixed-infections (Fig. 2) was generated using the UpSetR package and restructured manually to generate the side by side bar graphs for comparing two groups [52].

## Supplementary information

**Additional file 1: Table S1.** List of gene accession numbers downloaded from GenBank (NCBI) (https://www.ncbi.nlm.nih.gov/nucleotide/). **Table S2.** Demographic and clinical manifestations of DEC mono-infection in children hospitalized with diarrhoea.

## Data Availability

All data generated or analysed during this study are included in this published article and its supplementary information files.

## Abbreviations

DEC: Diarrhoeagenic *Escherichia coli*
LMICs: Low-middle income countries
ETEC: Enterotoxigenic *E. coli*
EAEC: Enteroaggregative *E. coli*
EPEC: Enteropathogenic *E. coli*
EIEC: Enteroinvasive *E. coli*
STEC: Shiga-toxin producing *E. coli*
HUS: Haemolytic uraemic syndrome
HCMC: Ho Chi Minh City
GEMS: Global Enteric Multicentre Study
CV%: Co-efficient of variance
MC: MacConkey agar
