# Correction: Novel multiplex real-time PCR assays reveal a high prevalence of diarrhoeagenic *Escherichia coli* pathotypes in healthy and diarrhoeal children in the south of Vietnam

**DOI:** 10.1186/s12866-022-02567-1

**Published:** 2022-07-14

**Authors:** Vu Thuy Duong, Le Thi Phuong Tu, Ha Thanh Tuyen, Le Thi Quynh Nhi, James I. Campbell, Pham Van Minh, Hoang Le Phuc, Tran Thi Hong Chau, Nguyen Minh Ngoc, Lu Lan Vi, Claire Jenkins, Iruka Okeke, Ellen Higginson, Stephen Baker

**Affiliations:** 1 The Hospital for Tropical Diseases, Wellcome Trust Major Overseas Programme, Oxford University Clinical Research Unit, Ho Chi Minh City, Vietnam; 2 Children’s Hospital 1, Ho Chi Minh City, Vietnam; 3 University of Medicine and Pharmacy at Ho Chi Minh City, Ho Chi Minh City, Vietnam; 4 Children’s Hospital 2, Ho Chi Minh City, Vietnam; 5 The Hospital for Tropical Diseases, Ho Chi Minh City, Vietnam; 6 National Infection Service, Public Health England, England, UK; 7 Department of Pharmaceutical Microbiology, Faculty of Pharmacy, University of Ibadan, Ibadan, Nigeria; 8 Cambridge Institute of Therapeutic Immunology & Infectious Disease (CITIID) Department of Medicine, Cambridge Biomedical Campus, University of Cambridge, Cambridge, CB2 0AW UK

**Correction: BMC Microbiol 20, 192 (2020)**

10.1186/s12866-020-01878-5

Following publication of the original article [1], the authors subsequently identified an error in one of the probe sequences. The probe used for the detection of *rfb*O157 gene in the EHEC multiplex PCR was incorrect. The correct sequence of the probe should be LCCyan500-TAGGACCGCAGAGGAAAGAGAGGAAT-BHQ1”.
