# Supplementary material for: Novel multiplex real-time PCR assays reveal a high prevalence of diarrhoeagenic Escherichia coli pathotypes in healthy and diarrhoeal children in the south of Vietnam
Source: BMC Microbiol. 2020 Jul 3;20:192. doi: 10.1186/s12866-020-01878-5 (PMC7333254; doi:10.1186/s12866-020-01878-5)
Supplement: Supplementary file 1 — Additional file 1: Table S1. List of gene accession numbers downloaded from GenBank (NCBI) (https://www.ncbi.nlm.nih.gov/nucleotide/). Table S2. Demographic and clinical manifestations of DEC mono-infection in children hospitalized with diarrhoea. [file 12866_2020_1878_MOESM1_ESM.pdf]

Supplementary data

Table S1. List of gene accession numbers downloaded from GenBank (NCBI) (https://www.ncbi.nlm.nih.gov/nucleotide/)

Gene	Accession number				
eltB	FN649417.1	AP010910.1	EU113252.1	S60731.1	AB011677.1
	J01646.1	FN822745.1	FJ407053.1	NC_009786.1	NC_014232.1
	NC_017640.1				
estA	AJ868113.1	FN822745.1	J03311.1	M18346.1	M18345.1
	AB702969.1	M29255.1	M34916.1	NC_014232.1	
bfpA	KJ641929.1	KJ020707.1	AB364244.1	FN391181.1	DQ388534.1
	KJ020711.1	KJ020697.1	AB364243.1	FM180569.1	
rfbE_O157	CP007592.1	CP008957.1	JQ907520.1	CP001368.1	AF163334.1
	CP010304.1	CP008805.1	JN578668.1	AF163335.1	
stx1	NC_002695.1	NC_011356.1	JX206444.1	EU754740.1	KM406321.1
	NC_013364.1	AP010953.1	JX161807.1	LM997224.1	JQ327854.1
	CP001925.1	GQ429158.1			
stx2	HF572917.2	KF932362.1	CP008805.1	CP006027.1	HF558443.1
	KM516099.1	LM997391.1	KJ158456.1	HF558445.1	HF558442.1
	AB854290.1				

Table S2. Demographic and clinical manifestations of DEC mono-infection in children hospitalized with diarrhoea

**Characteristics**	**Number of patients (%)**												
	**ETEC (n=34)**		**EAEC (n=170)**		**EIEC (n=116)**		**EPEC (n=137)**		**EHEC (n=20)**		**Negative (n=1,385)**		***p* value [a]**
Socio-demographic													
Male	18	(52.9)	117	(68.8)	70	(60.3)	82	(59.9)	16	(80.0)	852	(61.5)	0.118
Age in months, *median* [IQR]	11.2	[7.0-20.4]	11.5	[7.4-18.1]	14.8	[6.7-24.5]	12.4	[8.4-18.7]	15.6	[8.3-21.2]	9.1	[5.7-14.5]	0.388
Growth [b]													
Obese or overweight	0	-	15	(8.8)	11	(9.5)	13	(9.6)	0	-	129	(9.3)	0.321
Wasted or severely wasted	4	(11.8)	23	(13.5)	7	(6.0)	5	(3.7)	1	(5.0)	126	(9.1)	**0.013**
Type of diarrhoea [c]													**<0.001**
Non-bloody diarrhoea	23	(67.6)	142	(83.5)	63	(54.3)	98	(71.5)	15	(75.0)	812	(58.6)	**<0.001**
Bloody diarrhoea	8	(23.5)	23	(13.5)	46	(39.7)	33	(24.1)	4	(20.0)	368	(26.6)	**<0.001**
Persistent diarrhoea	3	(8.8)	5	(2.9)	7	(6.0)	6	(4.4)	1	(5.0)	205	(14.8)	0.414
Clinical manifestations													
Number of episodes per day, *median* [IQR]	5	[4-9]	6	[5-10]	6	[5-10]	6	[4-10]	5.5	[4-8]	7	[5-10]	0.284
Moderate and severe dehydration [d]	3	(8.8)	20	(11.8)	15	(12.9)	23	(16.8)	8	(40.0)	123	(8.9)	**0.010**
Abdominal pain	5	(14.7)	30	(17.7)	25	(21.6)	23	(16.8)	6	(30.0)	287	(20.7)	0.363
Fever (≥37.5°C at enrolment)	19	(55.9)	104	(61.2)	69	(59.5)	83	(60.6)	12	(60.0)	764	(55.2)	0.985
Vomit, *n* (%)	15	(44.1)	108	(63.5)	76	(65.5)	90	(65.7)	15	(75.0)	753	(54.4)	0.238
Haematology													
Neutrophil count (10^3/µL), *median* [IQR]	5.6	[3.3-9.3]	4.3	[2.3-6.6]	5.4	[2.9-9.4]	4.2	[2.5-6.9]	5.5	[3.5-7.8]	3.5	[2.2-5.7]	**0.014**
C-reactive protein (mg/L), *median* [IQR]	8.4	[5.0-31.0]	5.0	[1.0-19.3]	8.0	[5.0-40.2]	5.0	[1.3-12.0]	8.0	[6.0-15.9]	6.0	[4.0-21.4]	0.132
Treatment													
Low-osmolarity ORS	29	(85.3)	164	(96.5)	110	(94.8)	131	(95.6)	20	(100)	1,285	(92.8)	0.125
IV rehydration,	3	(8.8)	19	(11.2)	15	(12.9)	26	(19.1)	8	(40.0)	141	(10.2)	**0.005**
Antimicrobials,	30	(88.2)	123	(72.4)	101	(87.1)	109	(79.6)	16	(80.0)	1134	(81.9)	**0.027**
Fluoroquinolones, *n* (% of antibiotic treated) [e]	20	(66.7)	66	(53.7)	65	(64.4)	77	(70.6)	12	(75.0)	717	(63.2)	0.077
Zinc	29	(85.3)	148	(87.1)	100	(86.2)	118	(86.1)	20	(100.0)	1,243	(89.7)	0.488
Probiotics	22	(64.7)	123	(72.4)	89	(76.7)	99	(72.3)	16	(80.0)	957	(69.1)	0.631
Outcomes													
Hospital stay in days, *median* [IQR]	4	[3-5]	4	[3-6]	3.5	[3-6]	4	[3-6]	4	[3-6.5]	5	[3-7]	0.582
Improved and recovered after 3 days [f]	32	(94.1)	157	(92.4)	106	(91.4)	129	(94.2)	18	(90.0)	1,204	(86.9)	0.872

ORS: Oral Rehydration Solution; IQR: Inter-quartile range

[a] Comparison among DEC mono-infection using χ^2 test or Fisher's exact test for categorical data or Kruskal-Wallis test for continuous data (negative group was not included)

[b] Obese: weight for length z score >3SD in children age < 24months; BMI for age z score >3SD in children age ≥24months,
Overweight: weight for length z score >2SD in children age < 24months; BMI for age z score >2SD in children age ≥24months,
Wasted: weight for length z score <-2SD in children age < 24months; BMI for age z score <-2SD in children age≥24months,
Severely wasted: weight for length z score <-3SD in children age < 24months); BMI for age z score <-3SD in children age ≥24months)[34]

[c] Type of diarrhoea including non-bloody diarrhoea (diarrhoea with mucus, <14 days), bloody diarrhoea (diarrhoea with blood, <14 days), and persistent diarrhoea (diarrhoea with mucus and/or blood, ≥14days)

[d] Dehydration classified according to Integrated Management of Childhood Illness[35]

[e] Fluoroquinolones included ciprofloxacin and norfloxacin.

[f] Defined as "recovered" if patient had <3 passages of loose stool in the past 24 hours or "improved" if patient had less episodes of diarrhoea and less mucus and/or bloody in comparison to the condition of the patient at enrolment.
